# Woman, Her Diseases and Remedies; and a Treatment on Acute and Chronic Diseases of the Neck of the Uterus

**Published:** 1854-06

**Authors:** 


Art. IV.—*Woman, her Diseases and Remedies; and, a Treatise on Acute and Chronic Diseases of the Neck of the Uterus.* By Charles D. Meigs, M. D., &c., &c.

The first of these volumes is the third edition of a work which first appeared in 1847; the other is the report made by Prof. Meigs to the American Medical Association on the diseases of the uterus, and which makes a part of the 6th volume of the Society's transactions. We are glad that this treatise has been rendered more accessible to the profession by its publication in a separate form, for it is one which deserves to be far more extensively circulated and read than it is likely to be in the work in which it originally appeared. More than once we have had occasion to refer reproachfully to the neglect of the volumes annually emanating from our great medical Congress, by the body of the profession. The mass does not yet appear to have felt the leaven diffused through a limited circle by the Association; and if the report of Dr. Meigs on Uterine Diseases had remained locked up in its Transactions it would have been a sealed book to most practitioners.

The larger volume of which we have given the title above is already well known to our readers. It has been extensively read; it is, eminently, a readable book. The famous Dr. Johnson is said to have declared that one of the very few books which could induce him to quit his bed an hour or two before his accustomed time of rising in the morning was *Burton's Anatomy of Melancholy.* We should think that these Letters of Dr. Meigs on Woman might tempt even an indolent student to abridge his morning nap and to protract his studies far into the night. When first sent abroad the style of the author was severely criticised by many reviewers, as wanting in the elevation and dignity becoming a grave scientific subject. Dr. Meigs has carefully revised his third edition, and besides many additions made to its matter he has attempted to reform his manner of the vices complained of, and hopes by the time another edition shall be called for "to have rendered the style and language still less exceptionable." But while endeavoring to avoid what seemed low on the one hand, he has studied to escape from dulness on the other, keeping in mind the precept of Horace, "that the reader is to be gratified as well as instructed." "But shall people," he asks, "who desire to make a contribution to the art that has absorbed their whole existence, refrain from doing so from a fear of offending in the matter of their manner? Would that be American like? And shall everybody go out of the world making no sign?" He has made his sign, and every American physician owes him respect and admiration for it. His books are real contributions to medical science and art.

The treatise on the diseases of the neck of the uterus may be regarded as in some sort an appendix to his Letters on the Diseases of Woman, or more properly a continuation of that larger work. It is illustrated by numerous plates, many of them colored, and forms a volume of exceeding beauty. Dr. Meigs begins his report by some details of the methods of examining females. The operation of touching and the "metroscopic" one are both a "direful necessity," but the one, he thinks, "is not essentially more revolting to the feelings of the sufferer than the other." His remarks on this subject, as indeed his practical observations throughout the work, are judicious and just.

